# Diagnosis of epithelial ovarian cancer using a combined protein biomarker panel

**DOI:** 10.1038/s41416-019-0544-0

**Published:** 2019-08-07

**Authors:** Matthew R. Russell, Ciaren Graham, Alfonsina D’Amato, Aleksandra Gentry-Maharaj, Andy Ryan, Jatinderpal K. Kalsi, Anthony D. Whetton, Usha Menon, Ian Jacobs, Robert L. J. Graham

**Affiliations:** 1 Stoller Biomarker Discovery Centre and Manchester Molecular Pathology Innovation Centre, Division of Cancer Sciences, Faculty of Biology Medicine and Health, University of Manchester, Manchester, UK; 2 School of Biological Sciences, Queens University Belfast, Chlorine Gardens, Belfast, BT9 5DL UK; 3 Department of Pharmaceutical Sciences, University of Milan, Milano, Lombardy Italy; 4 MRC Clinical Trials Unit at UCL, Institute of Clinical Trials & Methodology, Faculty of Population Health Sciences, University College London, London, UK; 5 University of New South Wales, UNSW Australia, Level 1, Chancellery Building, Sydney, NSW 2052 Australia

**Keywords:** Biomarkers, Oncology

## Abstract

**Background:**

An early detection tool for EOC was constructed from analysis of biomarker expression data from serum collected during the UKCTOCS.

**Methods:**

This study included 49 EOC cases (19 Type I and 30 Type II) and 31 controls, representing 482 serial samples spanning seven years pre-diagnosis. A logit model was trained by analysis of dysregulation of expression data of four putative biomarkers, (CA125, phosphatidylcholine-sterol acyltransferase, vitamin K-dependent protein Z and C-reactive protein); by scoring the specificity associated with dysregulation from the baseline expression for each individual.

**Results:**

The model is discriminatory, passes *k*-fold and leave-one-out cross-validations and was further validated in a Type I EOC set. Samples were analysed as a simulated annual screening programme, the algorithm diagnosed cases with >30% PPV 1–2 years pre-diagnosis. For Type II cases (~80% were HGS) the algorithm classified 64% at 1 year and 28% at 2 years tDx as severe.

**Conclusions:**

The panel has the potential to diagnose EOC one-two years earlier than current diagnosis. This analysis provides a tangible worked example demonstrating the potential for development as a screening tool and scrutiny of its properties. Limits on interpretation imposed by the number of samples available are discussed.

## Background

The mortality from epithelial ovarian cancer (EOC), in the United Kingdom was 4227 in 2016.^1^ In the UK the 5 year survival rate drops from 90% for the 31% of cases that are diagnosed at stage I; to 22% for the 49% of cases diagnosed at stage III or IV.^1^ The non-specific nature of early EOC symptoms combined with the rarity of the disease presents a major barrier to increasing rates of detection of pre-clinical disease through the primary care setting. Achieving a mortality reduction in EOC will require a screening strategy capable of triggering intervention early enough to alter the natural history of ovarian cancer.

In the context of a screening programme cancers may be divided into indolent disease, patients with which tend to die of other causes; treatable disease which if caught and managed early may go into extended remission; and untreatable disease amenable only to management and palliative care.^2^ A successful screening programme must identify sufficient numbers of treatable cases for timely intervention, prior to their progression to untreatable disease, to outweigh the cost and associated harms of misdiagnosis of healthy individuals and unnecessary treatment of indolent cases.

Two screening trials for EOC based on a multimodal strategy involving the risk of ovarian cancer algorithm (ROCA),^3^ which determines risk of ovarian cancer by detecting a sudden rapid increase in CA125 level from baseline, have recently been published: the United Kingdom Collaborative Trial of Ovarian Cancer Screening (UKCTOCS) in low risk postmenopausal women,^4,5^ and United Kingdom Familial Ovarian Cancer Screening Study (UKFOCSS) in younger women with greater than 10% lifetime risk based on family history or gene mutation in BRCA1/2.^6^ UKCTOCS demonstrated that a multimodal screening arm, incorporating ROCA as a first-line and transvaginal ultrasound as a second-line test, was able to diagnose EOC at earlier stage than the control arm whilst UKFOCSS demonstrated a stage shift in cases diagnosed during screening programme versus cases diagnosed over a year after screening stopped. At the time of the initial UKCTOCS mortality report, although the early detection by screening had not yielded a significant reduction it demonstrated a trend toward mortality benefit and further follow up is underway.^4–6^ The need remains for a screening strategy which enables identification of treatable EOC cases leading to a mortality reduction.

A screening tool assigns a risk score to each participant. Every possible demarcation threshold on the range of risk scores represents a sensitivity-specificity pair which, combined with disease prevalence, defines a positive predictive value and a negative predictive value. Specificity and consequently positive predictive value depend on the false positive rate. The numbers of false positives must be kept low to minimise the burden on health services and healthy participants, but the majority of cases put to the diagnostic test will be healthy so even a low error rate will result in high absolute number of false positives. So, to make a screening tool clinically viable the threshold score must be raised, sacrificing sensitivity for specificity until false positive numbers fall to an acceptable level. The earliest most treatable cases are those to which a tool is least sensitive, and which will be missed on the first pass. Detecting these cases requires sensitive tests that retain the highest specificity enabling unambiguous diagnosis which permits rapid initiation of clinical investigation and progress to treatment. It is hoped that panels of complimentary biomarkers may offer improved specificity over single biomarker tests, as non-target diseases and benign conditions that dysregulate a single biomarker should have minimal impact on other members of the panel. Efforts to develop such a panel have not been successful to date.

Here, we describe the development of a panel using data from a subset of the prospectively collected serum samples spanning up to 7 years time-to-diagnosis (tDx), obtained in the course of the UKCTOCS trial. The 7-year tDx timespan permits the discovery of biomarkers in serum collected prior to clinical diagnosis of EOC; the period in which the asymptomatic but treatable early forms of EOC occur. It has previously been shown by a nested case-control study within the Prostate, Lung, Colorectal and Ovarian Cancer (PLCO) screening trial that a biomarker panel intended to supplement CA125 for EOC diagnosis^7^ showed no improvement over CA125 alone, greater than 6 months tDx.^8^ Recently proposed panels for EOC,^9^ and for multiple cancers including EOC^10^ which were also developed in clinically diagnosed cancers may suffer the same lack of sensitivity in pre-clinical samples as demonstrated by the PLCO study.

Our previously published work^11,12^ identified several biomarkers for EOC. The following analysis presents a screening tool based on the dysregulation of CA125 (uniprot: Q8WXI7) and the previously described Vitamin K-dependent protein Z (PROZ, uniprot: P22891), phosphatidylcholine-sterol acyltransferase (LCAT, uniprot: P04180) and C-reactive protein (CRP, uniprot: P02741) against a pre-disease baseline established for each individual patient. We demonstrate the tool’s utility in a simulated annual screening programme providing the basis to consider,^13^ with the addition of further validation studies, how this may translate to a screening programme.

## Methods

### Serum samples

UKCTOCS (International Standard Randomised Controlled Trial, number ISRCTN22488978; ClinicalTrials.gov NCT00058032) was given ethical approval (North West MREC 00/8/34). Trial design; subject consent and ethical oversight; sample acquisition and storage; and CA125 quantification are available elsewhere^3–6,14^ see Table S1 for baseline characteristics of UKCTOCS participants used within this study.

Samples were drawn from the multimodal arm of UKCTOCS (50640 women) which, at the time this study was initiated, yielded 19 Type I and 109 Type II EOC cases. For this study 49 cases were selected, comprising 30 Type II and 19 Type I (of which 10 were borderline) EOC cases, for histological classification see Table S2.^15,16^ An additional 31 control women were selected with no family history of EOC, no diagnosis of cancer during follow-up and were matched by age, regional collection centre and collection date to the Type II samples. The multiple serial serum samples of these 80 women spanning up to 7 years prior to diagnosis comprised a sample set of 482 distinct samples. Serum levels for LCAT (Cloud Clone Corp., Wuhan, Hubei, China), CRP (AbCam, Cambridge UK) and PROZ (AbCam) were available from our previous described work.^11,12^

### Scoring dysregulation

**Fig. 1:**
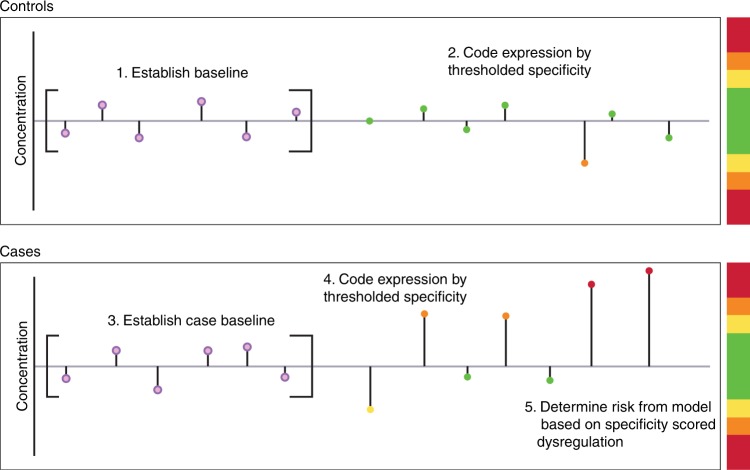
Graphical schematic illustrating the algorithm for calculating dysregulation scores. For controls, baseline expression is calculated as the mean of the earliest up to three samples more than 2 years tDx. Deviation from that baseline is then used to score biomarker dysregulation either by up- or down-regulation based on specificity thresholds calculated from the controls

All data analysis were performed using R.^17^ As controls do not have a time to diagnosis, for comparison with cases, samples from the control women were allocated a tDx value so that the last sample for each control was matched to the last sample from the matched case allowing temporal alignment of controls and cases. For each subject a baseline expression was estimated by taking the mean of up to the three earliest samples available for each subject drawn greater than 2 years tDx, this is the time interval where cases behaved as controls. For each sample, from each individual, absolute dysregulation from baseline was calculated. Specificity thresholds for dysregulation for each marker were set from the control data. For CA125, protein Z and LCAT this was so that specificity <80% was assigned score 0, 80–90% 1, 90–95% 2, 95–97.5% 3, 97.5–98% 4, 98–99% 5 and >99% 6, for CRP it was set to <70% 0, 70–80% 1, 80–90% 2, 90–95% 3, 95–99% 4, 99–99.5% 5, 99.5–99.9 6, and >99.9% 7 to give the model access to CRP’s wider range of specificities. See Fig. 1 for graphical schematic of the process.

### Model training and performance

A logit model was fitted to a training data set comprising Type II EOC samples less than 2 years tDx and control samples excluding three control women who developed cancer after study initiation. This encompassed 28 control and 25 case subjects, providing 118 control samples and 50 case samples, resulting in a total of 168 samples.

Model coefficients for each biomarker dysregulation score and significance of contribution to the model by the Wald test were tabulated and the odds ratio describing the fold change in risk associated with each increment in score was calculated for each marker from the exponential of the coefficient. Receiver operator curve analysis was performed using the pROC package for R.^18^

### Model cross-validation

To get an unbiased estimate of the predictive ability of the model we used two established methods for sample sets of this size in EOC research; Leave-one-out cross-validation and *k*-fold cross-validation. Leave-one-out cross-validation has been shown to be an efficient alternative to having separate sample sets for validation and it provides an unbiased estimate of the prediction accuracy. Leave-one-out cross-validation was performed by excluding each subject in turn from the training data set and re-training the model to confirm the stability of fitted coefficients. Further *k*-fold cross-validation was performed by excluding a tenth of the samples from the training set and re-training the model again to confirm the stability of fitted coefficients also enabling ROC analysis of predictions made against the excluded set as above. Standard deviations of the coefficients were calculated for each biomarker for each of these cross-validation strategies as a measure of the stability of the model to restricted sample sets.

### Simulated screening programme

The prospectively collected retrospectively analysed sample set enabled simulation of EOC cases progression through a simplified screening programme. The schema for the simulated programme was based on a simplified version of the multimodal screening arm of UKCTOCS.^14^ Since it is impossible to retrospectively apply the protocol of that study with its repeat samples, ultrasound scans and two-line strategy, a first-line annual screen relying on a blood test alone was simulated. The positive predictive value (PPV) is the probability that a woman with a given risk score actually has EOC which is the critical question for a clinician interpreting a test result. Positive predictive values (PPV) were calculated from the sensitivity-specificity pairs associated with each logit score obtained from ROC analysis of EOC cases (Type I and II) <2-years tDx vs all controls, combined with the prevalence of EOC in the unscreened arm of the UKCTOCS trial.^4^ The latest sample prior to the annual screen cut-off 1–4-years tDx, were classified severe (S), elevated (E) or intermediate (I) if they crossed PPV thresholds of 30%, 5% and 2.5% respectively, remaining samples were classified normal (N). The classifications are analogous to those set in the UKCTOCS trial, although with a much less sophisticated schema. UKCTOCS set elevated (E) or intermediate (I) at PPV thresholds of 0.2% and 0.02857%, respectively and introduced a severe (S) classification at a PPV threshold of 20% for women returning for a second line screen six weeks after an unsatisfactory first-line screen. This retrospective analysis cannot duplicate the conditions of the larger prospective UKCTOCS trial in which the ROCA algorithm was deployed in a multiple-step strategy incorporating ultrasound scans, so a direct comparison is impossible. The passage of EOC case samples through our simulated screening programme was visualised with a river plot using the riverplot package for R.^19^

### Algorithm implementation

For a step by step description of the diagnostic algorithm including look-up tables and model equations see Supplemental methods. Briefly; dysregulation scores are calculated as described above for each of the four proteins; the equation of the model is used to calculate the risk of ovarian cancer as a probability in the range 0–1; finally, the risk classification is determined from a table of thresholds based on the positive predictive value associated with the probability score.

## Results

### Model parameters and performance

Summary statistics for the model are presented in Table 1 in which the coefficients are the parameters of the model; the *p*-values indicated the significance of each biomarker’s contribution to the model by the Wald test; and the odds ratio describes the fold change in risk associated with each step up in dysregulation score for each biomarker. The marker, CA125, has the highest significance and makes the largest contribution to the odds ratio. The three additional markers have similar odds ratios to each other and the same order of magnitude as CA125 indicating they make a valuable contribution to the model. ROC analysis of the four biomarker panel model gave AUC 0.971 for EOC samples <1-year tDx vs all controls, 0.920 for EOC samples <2-years tDx and 0.848 for EOC samples 1–2-years tDx see Fig. S1 for ROC curves.

**Table 1:** Summary statistics for the model

Biomarker	Coefficient	*p*-value	Odds ratio	LOO Coeff SD	k-fold cross-validation coeff SD
Intercept	−3.5270	4.04e−11	0.0294	0.11	0.21
CA125	0.8217	6.45e−11	2.27	0.025	0.037
Protein Z	0.5345	8.72e−4	1.71	0.028	0.055
LCAT	0.3595	0.0211	1.43	0.024	0.059
CRP	0.3419	0.0260	1.41	0.043	0.065

Table showing, for each biomarker; the model coefficients *p*-value indicating significance of contribution to the model by the Wald test; the odds ratio, indicating the contribution of each additional step up on the dysregulation score, for that biomarker, to the risk attribution of the sample; leave-one-out (LOO) coefficient standard deviation, showing the stability of coefficients to exclusion of each subject and the *k*-fold cross-validation coefficient standard deviation, showing stability of coefficients to excluding 10% of samples from the model in turn

### Model cross-validation

To get an unbiased estimate of the predictive ability of the model we used two established methods for sample sets of this size in EOC research; leave-one-out cross-validation and *k*-fold cross-validation.^20–22^ The standard deviations of the coefficients from these models are presented in Table 1 and have a mean of 10%, indicating the coefficients are not highly dependent on the composition of the data set. Furthermore, each of the *k*-fold cross-validation models furnishes a set of predictions on samples excluded from the training set. ROC analysis of each of these test sets gave a mean AUC of 0.957 for EOC samples <1-year tDx vs all controls, 0.854 for EOC samples <2-years tDx and 0.774 for EOC samples 1–2-years tDx see Fig. S2 for all ROC curves. Together these results demonstrate that in this sample set risk of ovarian cancer is successfully modelled by the dysregulation of the panel of protein biomarkers.

### Sensitivity to Type I EOC cases

The four biomarker model was not trained against the Type I EOC cases. The Type I EOCs have different histology and are less aggressive than the Type II cases. Although not truly independent in the sense that they derive from a separate study they provide a challenge to the model trained against Type II cases. Sensitivity of the model to the Type I cases shows the model is not merely sensitive to the samples it was trained against but is finding a real signal. ROC analysis gave AUC 0.891 for EOC samples <1-year tDx vs all controls, 0.810 for EOC samples <2-years tDx and 0.705 for EOC samples 1–2-years tDx see Fig. S3 for all ROC curves. This shows the model trained in Type II cases is able to diagnose both Type I and Type II EOC cases and provides further confidence the model is not over fitted to the training set.

### Relationship between dysregulation scores and risk classification

The model takes as input dysregulation scores 0–6 for CA125, protein Z and LCAT and 0–7 for CRP. Considering risk classification attributed to different combinations of scores gives some insight into how the model might behave in a screening programme. The model input dysregulation scores would be as follows: {CA125; PROZ; LCAT; CRP} and the model results given as {model score; classification N,I,E or S}.

If this panel offers an improvement on the current test which is based on CA125, it would be expected to require contribution from more markers than just CA125 to attribute the highest risk scores. This is, in fact, the case as CA125 maximally dysregulated with no other biomarkers included is {6,0,0,0}→{0.80, E}. To attain a severe level classification requires the contribution of the maximum CA125 score with at least a single score on one of the other markers {6,1,0,0}→{0.87, S}; {6,0,1,0}→{0.85, S} {6,0,0,1}→{0.85, S}. The specificity of the test derives in part from the requirement that risk be indicated by multiple markers, by not detecting conditions known to elevate CA125 such as endometriosis. For this sample set higher EOC risk estimates generally resulted not from substantial dysregulation of a single marker in the panel but from lesser dysregulation of multiple panel members.

A successful panel would be expected to return a high risk of EOC for moderate dysregulation scores on each of the biomarkers in the panel. This is in fact the case as illustrated by the following score combinations: { 2, 3, 3, 3}→{0.86, S}; { 3, 2, 3, 2}→{0.85, S}; and { 3, 3, 2, 2}→{0.86, S}. Thus, scores for each individual marker, with specificity in the range of 90–97.5%, which would individually be of no use in a screen; combine to form a test that is highly specific, which retains sensitivity by drawing on the dysregulation of multiple markers.

The potential of CRP, which is known to elevate in many inflammatory events, to confound the screening tool with false positives is limited by the way the model has been constructed. The maximum score achievable by CRP dysregulation, with no contribution from other members of the panel, is 7. This would place an individual into the intermediate category {0,0,0,7}}→{0.24, I}. Thus, elevation of CRP alone would not trigger urgent intervention in a screening tool and the role of CRP in the model is to enhance the risk score afforded by the other more specific members of the panel.

### Simulated screening programme

**Fig. 2:**
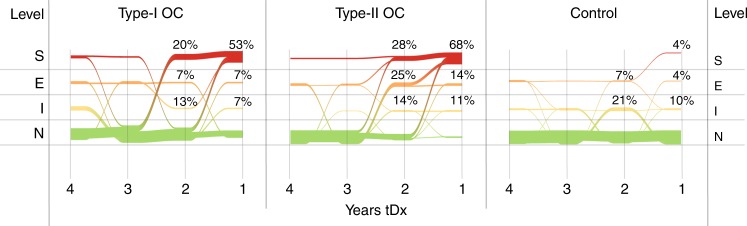
River plots showing the progress of women’s risk classification for years 4–1 tDx by taking the risk classification of the latest sample available prior to each annual screen cut-off. Plots based on the novel panel for Type I EOC cases (not used to train model) Type II EOC cases (used to train model) and controls may be compared. Risk classification elevates through intermediate (I), elevated (E) and severe (S) for the novel panel for both Type I and Type II cases whereas the majority of control women remain classified as normal. Coloured lines indicate risk classifications, normal (N) is green, intermediate (I) yellow, elevated (E) orange and severe (S) red

Assigning classifications severe (S), elevated (E), intermediate (I) or normal (N) to samples depending on the PPV associated with the sample enables simulation of a simplified annual screening programme to enable consideration of how the screening tool might be implemented in a screening programme. The progress of individuals through the screening programme is visualised as a river plot Fig. 2, which shows how classification of samples from women who developed Type I (not used to train model) or Type II (used to train model) EOC evolve over time. The visualisation shows the majority of women enter the simulated screening programme at 4-years tDx with samples classified as normal and subsequent samples show successively elevated risk towards diagnosis. For Type II EOC the novel panel assigns 64 and 28% of women to severe at 1 and 2-years tDx, respectively. For Type I EOC the novel panel assigns 53 and 20% of women to severe, at 1 and 2-years tDx, respectively. The controls show a majority at classification normal over the 4-year period, however, some controls (6 at year 4; 5 at year t3; 8 at year 2; 5 at year 1 tDx) were seen with classifications other than normal over this time. Within these samples there is a general pattern of risk estimates rising and then falling again, suggesting a repeat testing strategy like that deployed in UKCTOCS, might successfully eliminate them. There is a single control case that is classified elevated at 4, 3 and 2 years tDx, but which then rises to severe 1-year tDx. This case is more problematic and further investigation is required to understand how frequent cases like this might be and if strategies are available to deal with them.

**Fig. 3:**
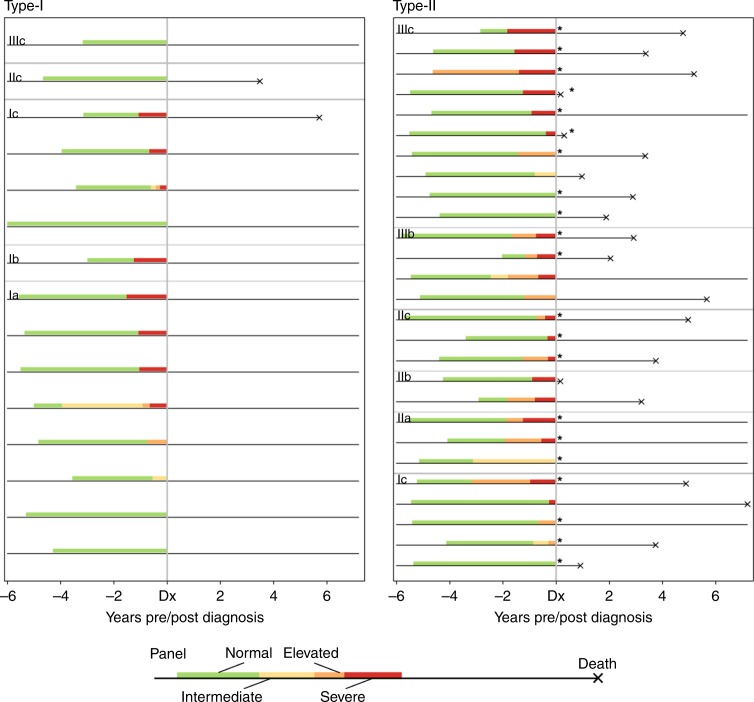
Each case is represented by a line from left to right across the graph, where disease-specific death was recorded post diagnosis this is indicated by terminating the line with a cross. Cases are separated by panel into Type-I and Type-II EOC and grouped within panel by stage at diagnosis. Coloured bars indicate the range of unbroken runs of samples prior to diagnosis, assigned incremental risk classifications of at least normal (N), intermediate (I), elevated (E) or severe (S). High grade serous cases, the most frequent form of the disease responsible for the highest mortality are indicated by a *

A further visualisation of each individual EOC case increasing risk classification from the panel is shown in Fig. 3 enabling direct comparison of risk scores for each woman. Cases are separated by panel into Type I and Type II and grouped within panel by stage at diagnosis. Coloured bars indicated the range of unbroken runs of samples prior to diagnosis assigned incremental risk classifications of at least normal (N), intermediate (I), elevated (E) or severe (S). Survival time post diagnosis gives further clues to the aggressiveness of each cancer.

The data also reveal features that add confidence to the model’s veracity. For example, Type II cases diagnosed at stage III are generally given risk classifications E or S at longer lead times to diagnosis than cases diagnosed at stages I and II, as would be expected if the women had harboured undiagnosed tumours for long enough for disease to reach the later stages. Over half the cases diagnosed at stage III were given risk classifications of S 1–2-years prior to diagnosis in the course of the trial, which invites speculation into how clinical outcomes for these women might have improved were the panel to have been available to them.

Of the two Type I case fatalities, one was not picked up prior to diagnoses by the panel, however, the other was classified S by the panel a year prior to diagnosis.

## Discussion

Ovarian cancer is a heterogeneous disease with tumour histology identifying multiple distinct types with differing prognosis, genetic markers and treatment regimens.^23^ It is possible that some of these ovarian cancers are so aggressive they are essentially untreatable and their successful identification in a screening trial may not reduce mortality. The challenge is to increase the identification of those treatable cases that do exist and to do so before they progress and become untreatable.

In this study of prospectively collected and retrospectively analysed samples, the tool presented above demonstrates an improved performance over CA125 interpreted by ROCA alone for early detection of ovarian cancer in the studied sample set. The four protein model was able to offer up to a 1–2-year lead time in diagnosis over that achieved for the cases in the UKCTOCS trail for both Type I and Type II cases. A direct comparison to the ROCA algorithm applied in the UKCTOCS trial and UKFOCSS study, is not possible retrospectively and the smaller sample set used here is likely to have resulted in an overestimation of the achievable PPVs. However, the signal identified in the protein panel is certainly real and warrants further investigation in independent sample sets both in Type II and Type I EOCs.

The simulated screening programme shows the potential for relatively subtle dysregulation, against patient baseline, of multiple biomarkers in combination to return a sensitive and specific assessment of EOC risk. Since either up- or down-regulation of each marker contributed to risk estimation it may be better to think of these makers not being directly mechanistically linked to the initiation and progression of disease, but as independent polls on dysregulation of several physiological systems. The degree and direction of dysregulation being determined by multiple interacting feedback mechanisms. There is a general lack of correlation between biomarker scores indicating their independence whilst the cases with low CA125 scores may still have high EOC probability scores due to contributions from other biomarkers showing how that independence contributes to sensitivity.

A caveat to the current model that should be borne in mind is that the number of controls (141 samples from 31 women) may underestimate the variability of expression of these proteins in women without ovarian cancer. Higher variability would reduce the specificity of the test, reduce the PPV of risk estimates and erode the confidence in diagnosis. Specificity is critical at two points in the analysis. The first is where dysregulation from baseline is scored by specificity thresholds derived from the controls. The second is in the simulated screening programme where PPV thresholds are set to classify subjects as normal, intermediate, elevated or severe. One likely reason that this study has underestimated variation is that there may be non-EOC conditions, much more frequently occurring than EOC, but sufficiently rare as to have been excluding from this control set, that affect PROZ, LCAT and CRP levels just endometriosis and the menstrual cycle do for CA125.^24,25^ Analysis of the longitudinal behaviour of these proteins in a much larger cohort of control women is required to obtain more precise estimates of specificity and PPV. In mitigation of this, however, the most concerning member of the panel in this regard (CRP) returns only intermediate risk if dysregulated alone. This indicates robustness to confounding conditions. Despite the important caveats above the investigation of the relationship between possible combinations of dysregulation score and risk estimate indicate that elevation of risk on a single biomarker, even CA125, does not return the highest risk estimates.

The strategy of developing a biomarker panel in the context of a simulated screening (Figs. 2 and 3) programme based on the UKCTOCS trial has focused analysis on the clinically critical positive predictive value, which has further enabled risk estimates from the tool to be related to potential care pathways. The panel and associated model developed pursuing this strategy is able to detect EOC in samples drawn from women 1–2-years prior to their diagnosis in the course of the UKCTOCS trial. This time period is expected to contain a high proportion of cases at a treatable stage where patients may benefit from a 90% rather than a 22% 5-year survival rate which would be of substantial clinical utility.

This analysis from the concept of taking markers as independent polls on physiological dysregulation (indicative of disease), through to a simulated screening programme, provides not just an abstracted statistical relationship between marker and disease status; but a tangible worked example accessible to oncologists. This study alone is not sufficient to justify deployment as a screening tool given the limitations of the analysis we have highlighted. The new panel does, however, have sufficient potential to justify a larger scale validation study and confirms the presence of EOC detecting signal from protein biomarker panels in early asymptomatic EOC.

## Supplementary information

Supplementary Methods

## Data Availability

UKCTOCS trial design; subject consent and ethical oversight; sample acquisition and storage; and CA125 quantification are available—Skates S.J. Ovarian cancer screening: development of the risk of ovarian cancer algorithm (ROCA) and ROCA screening trials. Int J Gynecol Cancer 2012. 22 Suppl 1, S24–26. Menon U., Gentry-Maharaj A., Hallett R., Ryan A., Burnell M., Sharma A. et al. Sensitivity and specificity of multimodal and ultrasound screening for ovarian cancer, and stage distribution of detected cancers: results of the prevalence screen of the UK Collaborative Trial of Ovarian Cancer Screening (UKCTOCS). Lancet Oncol 2009, 10, 327–340. Jacobs I.J., Menon M., Ryan A., Gentry-Maharaj A., Burnell M., Kals J.K. et al. Ovarian cancer screening and mortality in the UK Collaborative Trial of Ovarian Cancer Screening (UKCTOCS): a randomised controlled trial. Lancet 2016, 387, 945–956. Rosenthal A.N., Fraser L.S.M., Philpott S., Manchanda R., Burnell M., Badman P. et al. Evidence of Stage Shift in Women Diagnosed With Ovarian Cancer During Phase II of the United Kingdom Familial Ovarian Cancer Screening Study. J Clin Oncol 2017, 35, 1411–1420. *Protocol for the United Kingdom Collaborateive Trial of Ovarian Cancer Screening (UKCTOCS)*. Available from: https://www.ucl.ac.uk/womens-health/research/womens-cancer/gynaecological-cancer-research-centre/ukctocs/files/ukctocs_protocol71. Jacobs I.J., Skates S.J., MacDonald N., Menon U., Rosenthal A.N., Davies A.P. et al. Screening for ovarian cancer: a pilot randomised controlled trial. Lancet 1999, 353, 1207–1210. Protein Z, LCAT and CRP data are available—Russell M.R., Graham C., D’Amato A., Gentry-Maharaj A., Ryan A., Kalsi J.K. et al. A combined biomarker panel shows improved sensitivity for the early detection of ovarian cancer allowing the identification of the most aggressive type II tumours. Br J Cancer 2017, 117, 666–674. Russell M.R., Walker M.J., Williamson A.J.K., Gentry‐Maharaj A., Ryan A., Kalsi J.K. et al. Protein Z: a putative novel biomarker for early detection of ovarian cancer. Int J Cancer 2016 138, 2984–2992.
