# Supplementary material for: Diagnosis of epithelial ovarian cancer using a combined protein biomarker panel
Source: Br J Cancer. 2019 Aug 7;121(6):483–9. doi: 10.1038/s41416-019-0544-0 (PMC6738042; doi:10.1038/s41416-019-0544-0)
Supplement: Supplementary file 1 — Supplementary Methods [file 41416_2019_544_MOESM1_ESM.pdf]

Supplemental Methods

Algorithm Detailed Description

Introduction

This document describes in detail the algorithm applied in the paper. As outlined in the paper the algorithm was developed in a small sample set and the trained logit model described below is likely to require re-training as part of work in a larger data set.

Algorithm

1. Determine baseline expression of each of the proteins CA125 (uniprot: Q8WXI7); Vitamin K-dependent protein Z (PROZ, uniprot: P22891); Phosphatidylcholine-sterol acyltransferase (LCAT, uniprot: P04180); and C-reactive protein (CRP, uniprot: P02741) for the individual. This is calculated as the mean expression of up to the three earliest samples collected > 2 years prior to diagnosis. In a genuine screening program where a long time series was available samples taken 6-3 years prior to the test sample might be used.
2. Determine expression of each of the four proteins outlined above in the test sample.
3. Subtract the baseline expression from the test sample expression for each protein.
4. Allocate scores to each protein according to degree of dysregulation where the difference between baseline and current expression falls into ranges outlined in the table below:

Score	CA125 / U mL^{-1}	PROZ / ng mL^{-1}	LCAT / pg mL^{-1}	CRP / ng mL^{-1}
7				>27426
6	>6.84	>2470	>29900	16622-27426
5	6.29-6.84	2090-2470	26100-29900	9007-16622
4	5.98-6.29	1810-2090	25800-26100	5097-9007
3	5.48-5.98	1530-1810	23900-25800	2186-5097
2	4.24-5.48	1370-1530	20100-23900	1126-2186
1	2.59-4.24	1140-1370	16400-20100	521-1126
0	-1.56-2.59	-1040-1140	-16200-16400	-529-521
1	-1.56--1.91	-1040--1300	-16200--25100	-529--1016
2	-1.91--2.28	-1300--1610	-25100--31000	-1016--3036
3	-2.28--2.65	-1610--1710	-31000--31900	-3036--4200
4	-2.65--2.74	-1710--1730	-31900--32200	-4200--25337
5	-2.74--3.4	-1730--1780	-32200--35300	-25337--28674
6	<-3.4	<-1780	<-35300	-28674--29621
7				<-29621

5. Calculate the probability that the sample is OC positive from the following equation where $S_{protein}$ is the score for that protein from the table above:

$$\text{Probability} = \frac{1}{1 + e^{-[0.822S_{CA125} + 0.535\ \ _{PROZ} + 0.359S_{LCAT} + 0.342S_{CRP}]}}$$

6. Lookup the risk classification from the probability in the following table:

Probability Range	Risk Classification	Positive Predictive Value
0-0.2	N	< 2.5%
0.2-0.4	I	2.5-5%
0.4-0.8	E	5-30%
0.8-1	S	>30%

Supplemental Tables:

Table S1: Baseline characteristics of UKCTOCS participants used within this study.

Table S2: Case frequencies of histological classification and stage at diagnosis of of the primary ovarian cancers for this sample set (At the end of 2013 all of the samples used in this study underwent central review for confirmation of categorisation as Type I or Type II.)

Supplemental Figures:

Supplemental Figure S1: ROC curves for the model for time periods < 1 year to diagnosis, < 2 years to diagnosis and 1-2 years to diagnosis.

Supplemental Figure S2: All ROC curves generated during 10-fold k-fold cross validation.

The model was re-trained 10 times excluding a 10th of the samples from training set in each iteration. The ROC curves show the predictions made by the model produced in each iteration against the excluded data. As with the ROC curves presented for the model trained on the complete data set ROC curves are calculated for time period < 1 year to diagnosis, < 2 years to diagnosis and 1-2 years to diagnosis. For each time period the ROC curves from the 10 iterations have virtually the same performance and curve shape.

Supplemental Figure S3: ROC curves for the Type I samples, which were not used to train the model, and all controls for time periods < 1 year to diagnosis, < 2 years to diagnosis and 1-2 years to diagnosis.

Table S1:

	Median (25th - 75th centiles)							
	Control		Ovarian cancer					
			Overall		Type I		Type II	
	n=31		n=49		n=19		n=30	
**Age (years) at randomisation**	60.8 (58.4-65.8)		62.8 (58.7-67.3)		64.2 (58.9-69.9)		61.1 (58.7-65.5)	
**Years since last period at randomisation**	12.6 (6.6-18.2)		11.4 (5.7-18.2)		15.2 (8.1-22.6)		10.7 (4.0-16.1)	
**Duration of HRT use in those who were on HRT at randomisation (yrs)**	6.9 (5.8-11.7)		9.7 (4.8-13.0)		13.0 (10.7-13.9)		7.2 (3.3-11.6)	
**Duration of OCP use (yrs) in those who had used it**	10 (3-12)		6 (3-8)		5 (3-8)		6 (4-8)	
**Miscarriages (pregnancies < 6mths)**	0 (0-1)		0 (0-0)		0 (0-1)		0 (0-0)	
**No. of children (pregnancies > 6mths)**	2 (0-2)		2 (2-2)		2 (1-2)		2 (2-3)	
**Height (cms)**	162.6 (158.8-167.6)		162.6 (157.5-165.1)		162.6 (157.5-166.4)		162.6 (157.5-165.1)	
**Weight (kg)**	65.3 (62.6-74.0)		69.9 (62.6-78.9)		71.2 (66.7-79.2)		65.9 (61.7-75.8)	
	Number (%)							
	No.	%	No.	%	No.	%	No.	%
**Ethnicity:**								
**White**	30	96.8%	48	98.0%	18	94.7%	30	100.0%
**Black**	0	0.0%	0	0.0%	0	0.0%	0	0.0%
**Asian**	0	0.0%	0	0.0%	0	0.0%	0	0.0%
**Other**	1	3.2%	1	2.0%	1	5.3%	0	0.0%
**Missing**	0	0.0%	0	0.0%	0	0.0%	0	0.0%
**Hysterectomy**	1	3.2%	6	12.2%	2	10.5%	4	13.3%
**Ever use of oral contraceptive pill**	18	58.1%	21	42.9%	9	47.4%	12	40.0%
**Use of HRT at recruitment**	8	25.8%	9	18.4%	3	15.8%	6	20.0%
**Personal history of cancer***	0	0.0%	2	4.1%	1	5.3%	1	3.3%
**Personal history of breast cancer**	0	0.0%	1	2.0%	1	5.3%	0	0.0%
**Maternal history of ovarian cancer**	0	0.0%	1	2.0%	0	0.0%	1	3.3%
**Maternal history of breast cancer**	4	12.9%	4	8.2%	1	5.3%	3	10.0%

* includes those with personal history of breast cancer

Table S2:

	No.			
**Cancer type**	**Overall**	**Stage I**	**Stage II**	**Stage III**
**Type I**	**19**	**14**	**1**	**4**
**Borderline**	**10**	**10**	**0**	**0**
Serous	6	6	0	0
Mucinous	2	2	0	0
Endometrioid	2	2	0	0
**Invasive**	**9**	**4**	**1**	**4**
Low grade endometrioid	5	3	1	1
Clear cell	3	0	0	3
Adenocarcinoma	1	1	0	0
**Type II**	**30**	**7**	**8**	**15**
High grade serous	23	5	6	12
High grade endometrioid	3	1	1	1
Carcinosarcoma	1	0	0	1
Adenocarcinoma	3	1	1	1

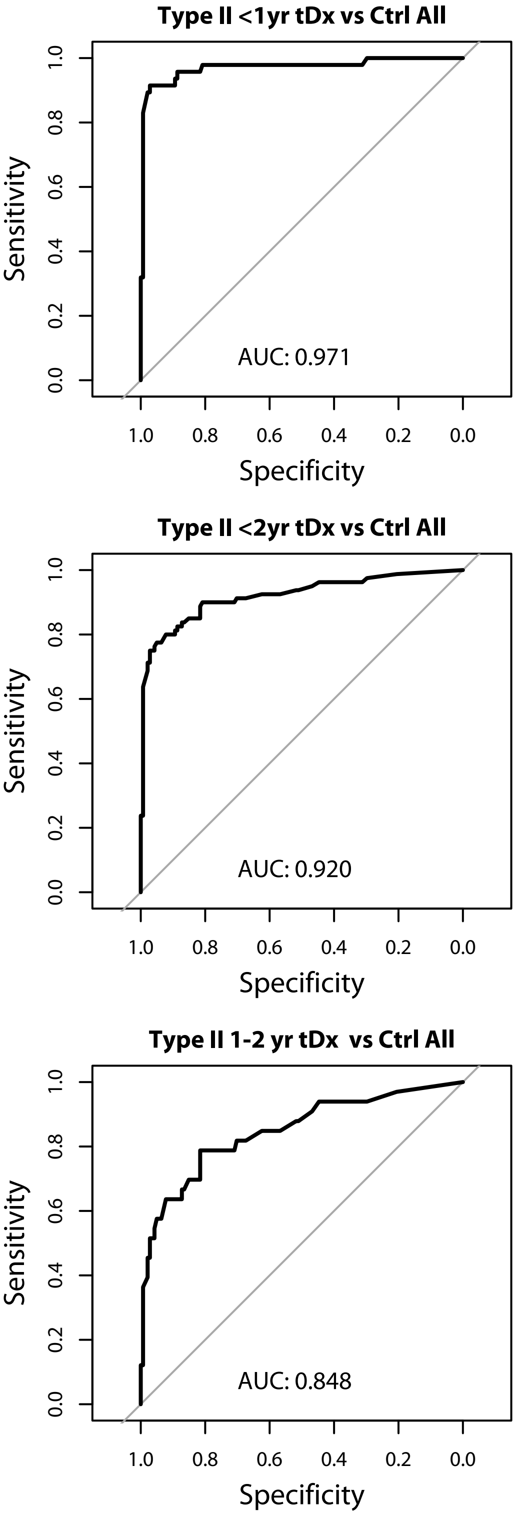
Type II <1yr tDx vs Ctrl All
Sensitivity
Specificity
AUC: 0.971
Type II <2yr tDx vs Ctrl All
Sensitivity
Specificity
AUC: 0.920
Type II 1-2 yr tDx vs Ctrl All
Sensitivity
Specificity
AUC: 0.848

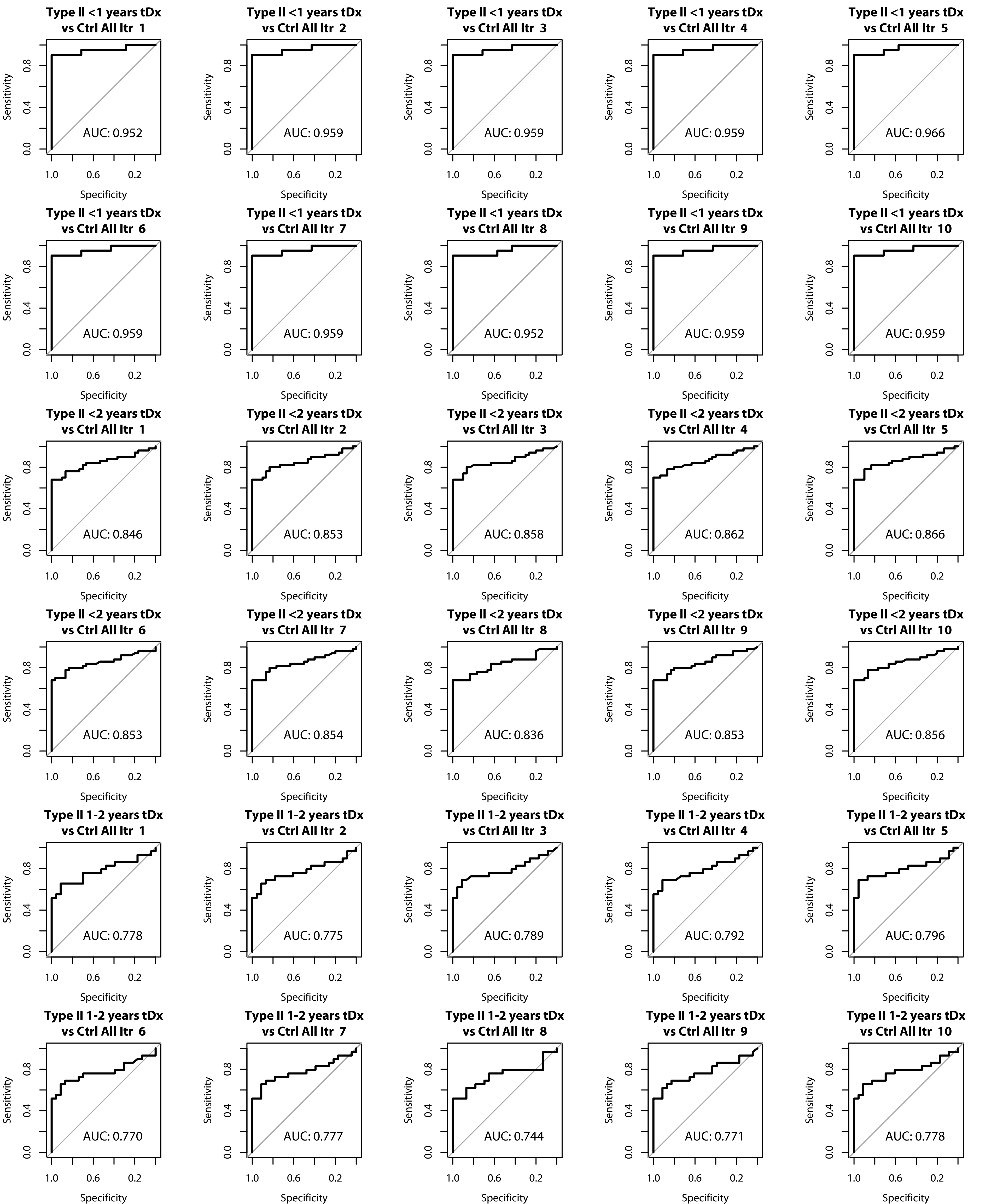

Type II <1 years tDx vs Ctrl All Itr 1
AUC: 0.952
Type II <1 years tDx vs Ctrl All Itr 2
AUC: 0.959
Type II <1 years tDx vs Ctrl All Itr 3
AUC: 0.959
Type II <1 years tDx vs Ctrl All Itr 4
AUC: 0.959
Type II <1 years tDx vs Ctrl All Itr 5
AUC: 0.966
Type II <1 years tDx vs Ctrl All Itr 6
AUC: 0.959
Type II <1 years tDx vs Ctrl All Itr 7
AUC: 0.959
Type II <1 years tDx vs Ctrl All Itr 8
AUC: 0.952
Type II <1 years tDx vs Ctrl All Itr 9
AUC: 0.959
Type II <1 years tDx vs Ctrl All Itr 10
AUC: 0.959
Type II <2 years tDx vs Ctrl All Itr 1
AUC: 0.846
Type II <2 years tDx vs Ctrl All Itr 2
AUC: 0.853
Type II <2 years tDx vs Ctrl All Itr 3
AUC: 0.858
Type II <2 years tDx vs Ctrl All Itr 4
AUC: 0.862
Type II <2 years tDx vs Ctrl All Itr 5
AUC: 0.866
Type II <2 years tDx vs Ctrl All Itr 6
AUC: 0.853
Type II <2 years tDx vs Ctrl All Itr 7
AUC: 0.854
Type II <2 years tDx vs Ctrl All Itr 8
AUC: 0.836
Type II <2 years tDx vs Ctrl All Itr 9
AUC: 0.853
Type II <2 years tDx vs Ctrl All Itr 10
AUC: 0.856
Type II 1-2 years tDx vs Ctrl All Itr 1
AUC: 0.778
Type II 1-2 years tDx vs Ctrl All Itr 2
AUC: 0.775
Type II 1-2 years tDx vs Ctrl All Itr 3
AUC: 0.789
Type II 1-2 years tDx vs Ctrl All Itr 4
AUC: 0.792
Type II 1-2 years tDx vs Ctrl All Itr 5
AUC: 0.796
Type II 1-2 years tDx vs Ctrl All Itr 6
AUC: 0.770
Type II 1-2 years tDx vs Ctrl All Itr 7
AUC: 0.777
Type II 1-2 years tDx vs Ctrl All Itr 8
AUC: 0.744
Type II 1-2 years tDx vs Ctrl All Itr 9
AUC: 0.771
Type II 1-2 years tDx vs Ctrl All Itr 10
AUC: 0.778
Sensitivity
0.0
0.4
0.8
Specificity
1.0
0.6
0.2

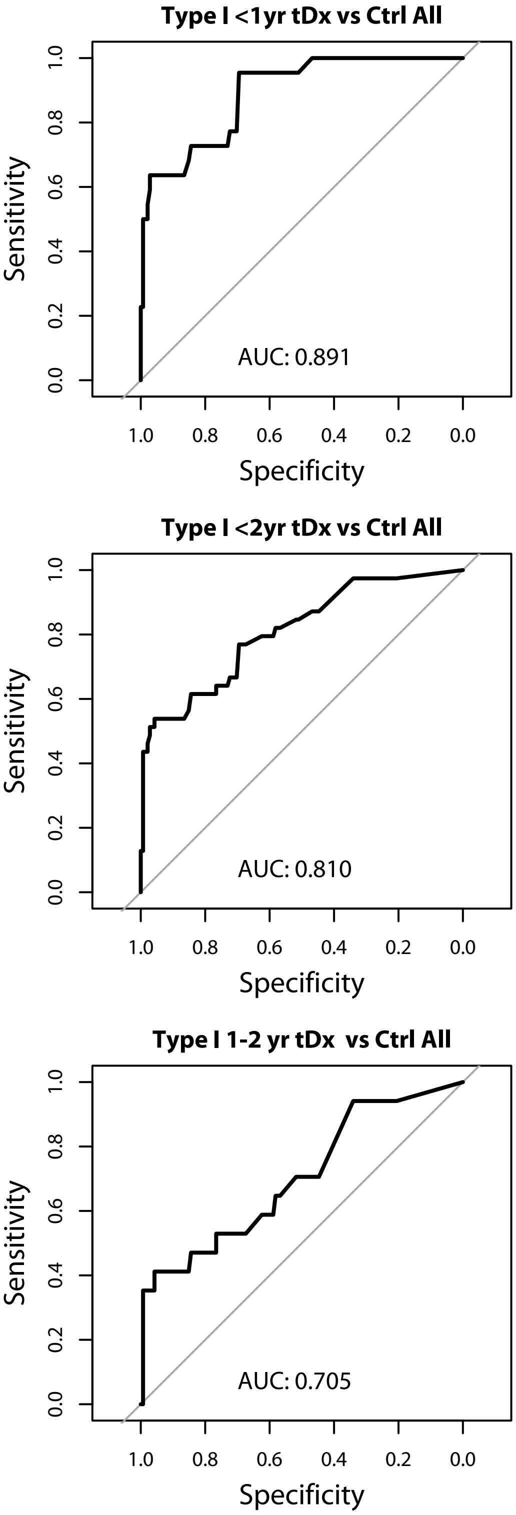

Type I <1yr tDx vs Ctrl All
Sensitivity
Specificity
1.0
0.8
0.6
0.4
0.2
0.0
AUC: 0.891
Type I <2yr tDx vs Ctrl All
Sensitivity
Specificity
1.0
0.8
0.6
0.4
0.2
0.0
AUC: 0.810
Type I 1-2 yr tDx vs Ctrl All
Sensitivity
Specificity
1.0
0.8
0.6
0.4
0.2
0.0
AUC: 0.705
